# An Alternative Approach in Anatomy Education: Design of a Learning Environment Based on Artificial Intelligence‐Supported Virtual Manipulatives and Investigation of Its Effectiveness

**DOI:** 10.1002/ca.70008

**Published:** 2025-07-29

**Authors:** Gunes Bolatli, Salih Birisci, Zafer Bolatli

**Affiliations:** ^1^ Department of Anatomy, Faculty of Medicine Yalova University Yalova Turkey; ^2^ Deparment of Computer Education and Instructional Technology Bursa Uludag University Bursa Turkey

**Keywords:** anatomy education, artificial intelligence, ChatGPT, simulation, virtual manipulative

## Abstract

Given the challenges of teaching and learning anatomy, it is vital to explore tools that enhance knowledge and understanding. In line with this need, this study aimed to develop a web‐based learning module with ChatGPT‐based artificial intelligence‐supported virtual manipulatives to enhance anatomy education and assess its efficacy. The ADDIE model was adopted for the instructional design process. By employing a mixed‐method research design, the effects of this learning module on the students' academic achievement, cognitive load, retention, and perceptions were analyzed. In the quantitative research phase, a quasi‐experimental pre‐ and posttest design was used to examine the effects of academic achievement and cognitive load, using the academic achievement test and cognitive load scale. Qualitative data were gathered via an interview form developed by the researchers. The results of the research demonstrate that the instructional design model that incorporates artificial intelligence‐based virtual manipulatives, is an effective approach to learning anatomy, with a notable impact on academic achievement and a reduction in cognitive load. While the students' perceptions of their educational experiences showed that the usability of the web module was well‐received and ChatGPT was adopted as a study companion, both positive and negative comments were made regarding the use of virtual manipulatives and ChatGPT. This study suggests that AI and virtual manipulatives may offer a promising alternative to traditional learning, providing an innovative framework for teaching and learning of anatomy.

## 1 Introduction

Anatomy is one of the introductory courses in all health sciences departments. It constitutes an important step in health sciences education, preparing students to move on to clinical sciences by providing learning about the structure and functioning of the human body. Inadequate anatomy education can lead to incorrect diagnoses and applications. Therefore, it is considered an integral part of quality health sciences education (Polat et al. 2024). Anatomy education has a theoretical and practical course structure. While theoretical courses are taught face‐to‐face in classroom environments, practical training is taught using concrete models in the form of models and cadavers in the laboratory (Mohapatra et al. 2015). However, several significant problems related to practical training exist, including as high costs, lengthy time requirements, and limited cadaver availability. These problems, especially in practical training, have enabled the discovery of innovative and complementary tools in anatomy teaching (Bolatlı and Bolatlı 2023).

The anatomy course contains numerous details that need to be learned, making it open to rote learning. The subject of the study is important for understanding the anatomy of the nervous system, comprehending its function, and applying this knowledge in clinical practice. However, students often find it difficult to grasp this topic (Iwanaga et al. 2021). Many students require alternative educational resources beyond printed materials to achieve success. Instructional technologies are intriguing as they help establish a balance between traditional and technological teaching methods (Zargaran et al. 2020). Two‐dimensional (2D) or three‐dimensional (3D) visual technologies developed primarily to facilitate learning anatomical structures provide many advantages for students compared to classical methods. Creating 3D visuals is an essential alternative for students with difficulty in anatomy education. It has been observed that studies with 3D visuals facilitate anatomy education and increase perception and satisfaction (Hu‐Au and Lee 2017). At the same time, simulations using 3D visuals reduce the learner's cognitive load and increase academic success (Silen et al. 2022).

Among the reasons for the increased use of technological methods, anatomy teaching is likely to be adaptable to various kinds of technology (Owolabi and Bekele 2021). It can be improved through the use of technology (Lee 2023). It is also known that students' learning habits have greatly improved in our century, and they are very interested in technology (Owolabi and Bekele 2021). Although studies on the use of technology in anatomy education exist, reaching healthy conclusions about its educational value seems complicated due to the lack of well‐designed, randomized, controlled studies in educational sciences (Bolek et al. 2021). Since the traditional subject‐based curriculum is content‐oriented and not evidence‐based, it creates disadvantages for students (Potu et al. 2013). These disadvantages affect students' understanding of the fundamentals of anatomical concepts, leading to a decline in their academic performance. In this context, there is a need to evaluate the effectiveness of the designed learning techniques, considering learning strategies in anatomy teaching (Abdellatif et al. 2022). At the same time, educators need to take advantage of the opportunities offered by technology to meet the changing learning needs of the new generation (Bolatli and Kizil 2022).

## 2 Conceptual Framework

Simulation can be defined as the imitation of the real form of an object, event, or situation in the world (Snir et al. 2003). This new method provides a safe and interactive environment for learning and teaching. In this context, the incorporation of simulations in the educational process has been demonstrated to enhance learner performance and mitigate errors. It also offers self‐regulated opportunities for learners to understand and transfer knowledge to understand concepts better (Al‐Elq 2010; Karbasi and Niakan Kalhori 2020). The simulation techniques are used extensively in many fields, including healthcare, education, and air defense. In healthcare, simulation techniques in controlled conditions enhance or refine doctor–patient interactions. Simulations, which can be developed within the scope of the opportunities offered by digital‐based environments, can be classified as computer programs that will allow students to directly experience situations that may be dangerous or impossible to reach in real life (Fink et al. 2021). In other words, the simulation technique is a virtual manipulative technique that evokes or replicates essential aspects of the real world in a fully interactive way (Torres et al. 2014). Virtual manipulatives, such as simulations, can be used to explore and learn about concepts or systems.

When Moyer et al. first described virtual manipulatives, they referred to them as web‐based, interactive learning tools in which objects can be moved with a computer mouse (Moyer et al. 2002). Virtual manipulatives can be categorized as new‐generation educational tools that bridge the gap between concrete and abstract learning experiences (Manches 2011). The term *virtual manipulatives*, as used in this study, refers to interactive 3D anatomical models that enable learners to manipulate, rotate, zoom, and dissect anatomical structures in a digital environment. These tools offer a dynamic and immersive learning experience that supports anatomical understanding and reasoning (Bartoletti‐Stella et al. 2021). Unlike traditional videos or 2D static demonstrations, virtual manipulatives let students actively interact with anatomical content, simulating the benefits of hands‐on dissection (Pettersson et al. 2023; Jang et al. 2017). However, for simulation or virtual manipulativetype tools to be effective during learning, they should possess several key features that should be given importance in their design. By incorporating these features with the multimedia design principles, they can create effective learning materials that enhance students' comprehension, participation, and overall learning (Mayer 2009). By integrating these principles into educational materials, educators can create compelling learning experiences for their students and foster better understanding and retention of knowledge. Another purpose of including these principles is to manage the cognitive load, which refers to the learner's mental effort during learning, and to eliminate or reduce the elements that challenge student's limited cognitive capacity (Mayer 2009; Sweller 2010). It is important to consider the role of an interactive, personalized, and adaptive learning environment in facilitating these transactions, where students engage with digital content while receiving real‐time feedback and guidance. This innovative approach combines immersive technologies with interactive learning, fostering a deeper understanding of anatomical structures. This situation can become possible within the possibilities offered by artificial intelligence, among today's developing technologies (Zhao et al. 2024).

Artificial intelligence is a field of computer science that focuses on solving cognitive problems such as learning, problem‐solving, and pattern recognition, which are usually related to human intelligence (Fitria 2021). ChatGPT, developed by OpenAI, an artificial intelligence company, is defined as an artificial intelligence model used in natural language processing (NLP) (Castelvecchi 2022). Its main goal is to interact with people in real‐time text‐based chats by generating human‐like responses and communicating in natural language (Fitria 2023; Atlas 2023).

AI models, including ChatGPT, have the potential to dramatically transform the way teaching, learning, and research are conducted in higher education. The accessibility and use of text‐generation tools such as ChatGPT are also increasing (Zhao et al. 2024). These models offer several advantages, such as assisting students in their studies, aiding researchers in information retrieval, and providing a natural language interface for educational resources (Atlas 2023). AI tools like ChatGPT provide immediate feedback and personalized learning experiences, increasing student engagement and promoting independent learning (Leng 2024). On the other hand, AI tools respond to student input in real time by adjusting parameters or presenting new challenges, ensuring that the manipulative remains engaging and educational (Taranikanti and Davidson 2023).

## 3 Theoretical Background and Research Questions

One of the challenges of learning science is determining how to present materials to help people learn (Mayer 2009). For this reason, instructional design (ID) models have been developed to help learning. Unlike normal (descriptive) theories, ID models are design‐based and allow for proper guidance of learners (Reigeluth 2013).

ID models are intended to assist educators in ensuring that they are delivering the correct content in an appropriate manner (Piskurich 2015). According to Wang and Hannafin, design‐based research is “a systematic yet flexible methodology based on collaboration between researchers and practitioners in the real world, aiming to improve educational practice through iterative analysis, design, development, and implementation, leading to context‐sensitive design principles and theories” (6). ADDIE (Analysis, Design, Development, Implementation, and Evaluation) is an ID model used in this methodology (Wang and Hannafin 2005). Many IDs and educational programmers widely use the ADDIE model to develop instruction and training programs. In the ADDIE model, each step has an outcome that strengthens the next step, and this progression does not require a linear sequence (Spatioti et al. 2022). Learning objectives set in the analysis phase may require additional work to deliver in the development phase; in such cases, the objectives must be adjusted. Practical difficulties encountered during the initial implementation of the learning program can be addressed by incorporating necessary elements during the design or development phases (Cheung et al. 2021).

Artificial intelligence can play an essential role in creating personalized learning experiences in the field of education. Educators can use this technology to effectively design learning experiences by focusing on students' needs and preferences (Bozkurt 2023). Personalized learning refers to adapting pedagogy, curriculum, and learning environments to meet students' learning needs and desires. This approach aims to increase students' learning potential while encouraging their active participation in the learning process (Kışla and Şahin 2015). AI‐supported personalized learning can analyze students' preferences and recommend appropriate content and learning strategies (Vorobyeva et al. 2025). These tools, known as Generative AI (GenAI) include ChatGPT, Gemini, Claude, and others have the potential to facilitate personalized learning, content creation, and offer enhanced reasoning capabilities, factual precision, and multimodal interaction features. ChatGPT is a generative pretrained transformer (GPT) based on NLP (Kar et al. 2023) that understands text and voice inputs and reproduces outputs. Although ChatGPT‐3.5 was released in late 2022, and more advanced versions such as ChatGPT‐4.0 are currently available, this study employed ChatGPT‐3.5 since its practical accessibility and alignment with the study's pedagogical objectives. Incorporating ChatGPT into anatomy education can provide new opportunities for personalized learning experiences and improved teaching and learning methods, especially in the face of constraints such as limited access to cadaveric material (Pandurangam et al. 2024). Resent research have shown that ChatGPT‐based GenAI models can enhance academic achievement by integrating them into anatomy education and can provide knowledge of anatomical concepts, useful for students and educators (Leng 2024; Saluja and Tigga 2024). In addition to these advantages, studies have also identified disadvantages of ChatGPT in anatomy education, including the generation of incorrect information and performance (Chytas et al. 2025). In studies comparing student responses with those provided by ChatGPT, it has been reported that ChatGPT demonstrates a higher accuracy rate (Talan and Kalınkara 2023); however, among various scientific disciplines, anatomy is the subject in which its accuracy rate is the lowest (Gencer and Gencer 2024). In a comprehensive analysis, it was stated that ChatGPT is not yet effective enough to play an active role in anatomy education; however, it should be re‐evaluated in the future if improvements are made over time (Chytas et al. 2025).

Traditional anatomy is a rote‐based course that fails to encourage students to learn (Champion et al. 2018). It is claimed that the use of AI technology in health sciences education will have a positive impact on the learning process (Bayne 2015; Botrel et al. 2015). The interactive nature of virtual manipulatives facilitates more profound understanding and retention of complex concepts, while the adaptability and real‐time feedback that AI provides ensure students are always working within their optimal cognitive capacity. For this reason, this study aims to design an artificial intelligence‐supported virtual manipulative‐based learning environment for anatomy education and to examine the cognitive load on students and its effect on academic achievement to evaluate the effectiveness of this learning environment. In this context, answers to the following research questions will be sought.

- Does a ChatGPT‐supported virtual manipulative learning environment affect students' academic achievement?
- Does a ChatGPT‐supported virtual manipulative education environment affect students' cognitive load?
- Does a ChatGPT‐supported virtual manipulative education environment affect students' retention test scores?
- What are students' opinions about ChatGPT‐supported virtual manipulative education environments?

## 4 Methodology

### 4.1 Research Design

This study adopted a mixed‐method research design (Vorobyeva et al. 2025) to evaluate the effectiveness of the instructional approach developed for anatomy education. Specifically, an explanatory sequential design was implemented, integrating quantitative and qualitative methodologies for data collection and analysis (Kar et al. 2023). The process commenced with the collection and analysis of quantitative data to assess students' academic achievement, cognitive load, and learning retention. Subsequently, qualitative methods were employed to gain an in‐depth understanding of students' reactions and experiences during the implementation process.

### 4.2 Participants

**FIGURE 1:**
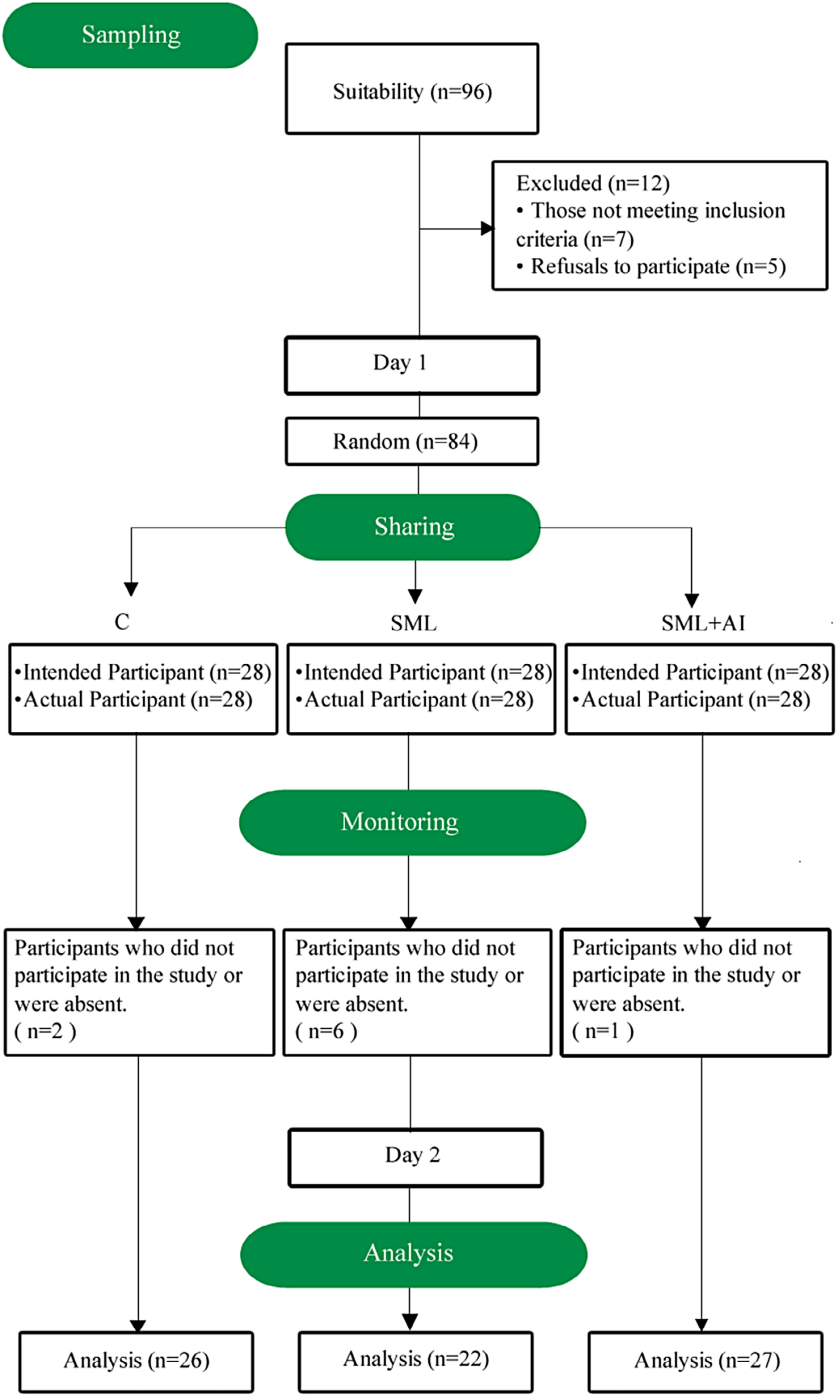
An example of the study flowchart is based on the CONSORT 2010 flow diagram (Moher et al. 2012).

The study group consisted of 75 junior nursing students in the northern Marmara region of Türkiye. The criterion sampling method (Pandurangam et al. 2024) was utilized to select participants, ensuring that students who had not previously engaged in anatomy or simulation‐supported training were included. Additionally, students were required to consent to take part in the study. By the nature of the study, the student groups were randomly divided into three groups: control (C), where traditional teaching took place, simulation‐supported teaching (SML), and simulation + artificial intelligence‐supported teaching (SML + AI). Accordingly, there were 26 students (19 female and 7 male) in C, 22 students (17 female and 5 male) in SML, and 27 students (21 female and 6 male) in the SML + AI group (Figure 1).

## 5 Measures

### 5.1 Anatomy Achievement Test (AAT)

The AAT was developed by the researcher to cover the subjects of Anatomy and Nervous System education. AAT was applied before and after the experimental study, as well as to evaluate the retention in learning. While various techniques, such as observations or the completion of applied tasks, can be employed to measure academic achievement, this research was based on the outcomes yielded by the assessment of achievement through test–retest comparison. The questions in AAT, prepared according to Bloom's taxonomy criteria, were at the level of recall and comprehension, and consisted of 20 in total. Four were visual questions based on the atlas picture, three were open‐ended, and the other 13 were multiple‐choice. The questions were grouped by category: general anatomy (Bolatlı and Bolatlı 2023), central (Lee 2023), peripheral (Zargaran et al. 2020), and nervous system diseases (Bolatlı and Bolatlı 2023). Experts examined the questions in the developed test in the field, and their reliability was ensured. Each question in the test, which students were given 30 min to complete, is worth 5 points, and the total score ranges from 0 to 100. To assess internal consistency, Cronbach's alpha was computed and found to be *α* = 0.78, indicating acceptable reliability for educational assessments (George and Mallery 2003). This reliability score supports the consistent measurement of students' anatomical knowledge and contributes to the credibility of the findings derived from pretest, posttest, and retention‐test comparisons.

### 5.2 Cognitive Load Scale (CLS)

The CLS, developed by Paas and Van Merriënboer (1993), measured the cognitive load students experienced while performing tasks during anatomy teaching. This single‐item scale (e.g., How much effort did you put into learning the concepts of the nervous system?) was scored on a nine‐point scale (1 = *very little*, …, 9 = *very very much*), with a minimum score of 1.00, a midpoint of 5.00, and a maximum score of 9.00. Participants who scored below five points were cognitively unloaded, while those who scored above five were cognitively loaded. The Cronbach's alpha value used for the reliability of the scale adapted into Turkish by Kılıç and Karadeniz was calculated as 0.78. Özbay and Seferoğlu calculated the Cronbach's alpha value as 0.89 in another study (Özbay and Seferoğlu 2023; Kılıç and Karadeniz 2004). In the present study, Cronbach's alpha reliability coefficient was calculated as 0.79.

### 5.3 Semistructured Interview Form

This interview aimed to assess the views of SML + AI group students on the anatomy learning environment after two week. Students were asked to write their opinions about AI‐supported learning, virtual manipulatives, their impact on anatomy learning, and the suitability of these approaches in the classroom. Close‐ended questions (e.g., agree‐disagree or Likert‐type scales) or interviews were the options to be used; however, semi‐structured open‐ended question responses should be used to enhance, confirm, or refine the views better, as the quantitative data (Yıldırım and Şimşek 2006). This situation allows the data source to be confirmed and validated than data obtained through questionnaires (Karasar 2006). Subsequently, the prepared interview form was submitted for review to both experts in the fields of anatomy education and instructional technologies at the university. The final form comprised five questions, allowing students to express their opinions in written form (e.g., “What are your views on artificial intelligence and virtual manipulative assisted instructional design?”).

### 5.4 Procedure

The nervous system was chosen as the instructional focus based on an analysis of previous course records, expert feedback, and relevant literature. The participants were first‐year nursing students, for whom the nervous system is typically taught as 2 h of theoretical instruction and 2 h of practical sessions per week, delivered over 2 weeks (8 h in total). All groups received 4 h of theoretical instruction; however, the 4h practical sessions differed across groups: Group C engaged with physical models and cadavers in the anatomy lab, while the SML and SML + AI groups utilized computer‐based simulations in a classroom environment. In contrast, the SML + AI group leveraged ChatGPT, embedded within the simulation platform, to ask questions and receive immediate AI‐generated feedback. The same instructor facilitated the teaching process across all three groups to ensure consistency.

**FIGURE 2:**
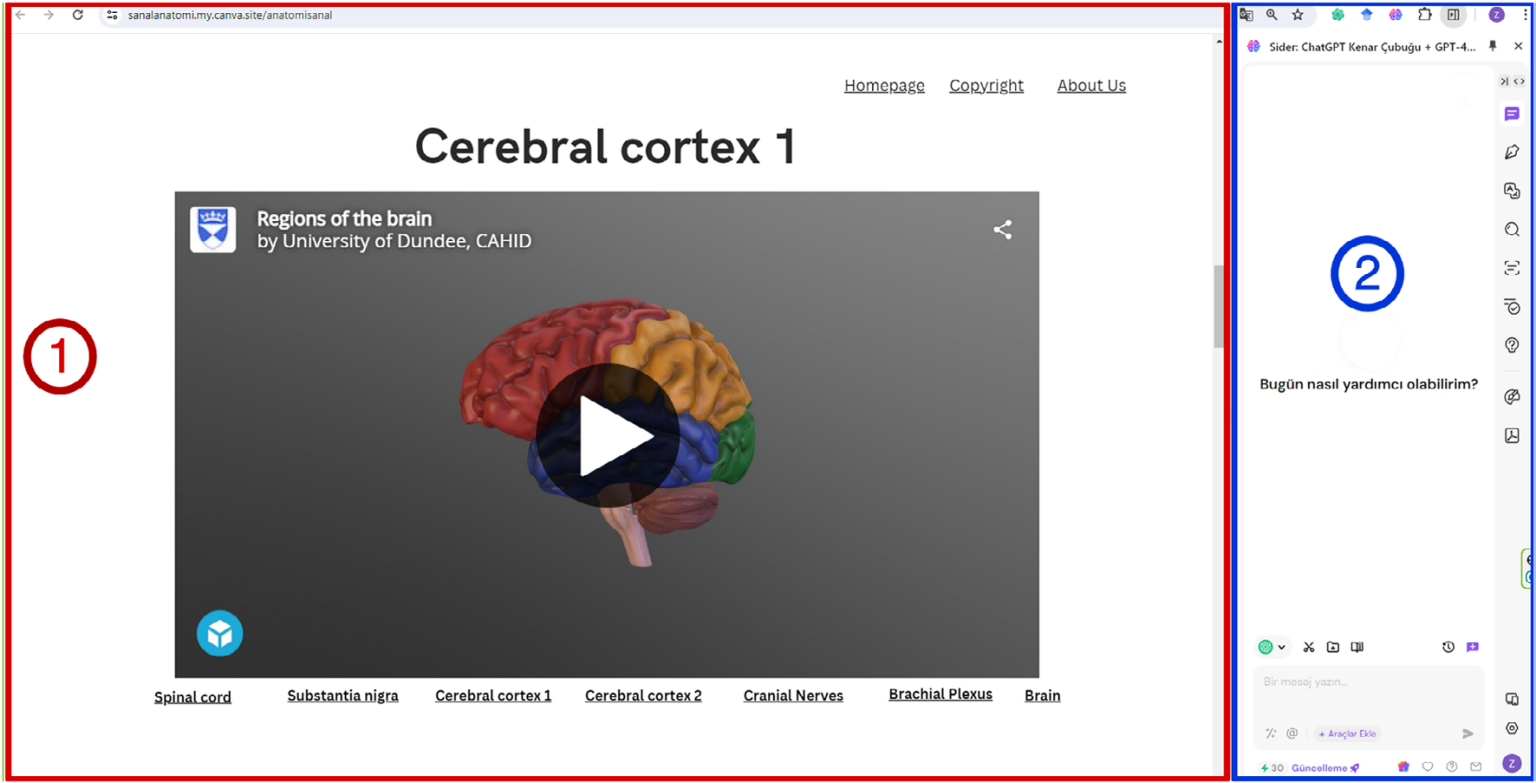
Virtual manipulation and artificial intelligence support the view of the related web page.

Before the intervention, the AAT was administered as a pretest. Instructional materials included seven 3D virtual manipulation models (e.g., medulla spinalis, cortex cerebri, and cranial nerves), sourced from AnatomyTOOL and Sketchfab under Creative Commons licensing. On the other hand, ChatGPT 3.5 was utilized within the scope of artificial intelligence in the developed system, and the Sider (Side Bar) feature, a Chrome plug‐in, was integrated. It is used as a parallel system, allowing SML + AI students to ask as many quick questions as they want and receive immediate answers. This ID was integrated into a web system (https://sanalanatomi.my.canva.site/anatomisanal) to provide access to virtual learning materials featuring two main components: 3D virtual manipulatives on the left and ChatGPT interactions on the right (Figure 2). In the other experimental group (SML), the same website was used without the ChatGPT sidebar plug‐in. In groups SML and C, the instructor answered students' anatomical questions directly during the sessions.

A pilot test involving the SML and SML + AI groups assessed the usability of the system. Students accessed the simulations on their computers, exploring features such as ChatGPT‐assisted interactions. Furthermore, SML + AI students also familiarize themselves with the ChatGPT‐integrated platform. Initial difficulties in forming effective AI prompts were mitigated by providing sample keywords and detailed guidance. It was stated that they could access detailed information about the virtual model of the relevant nervous system by using the essential questions they wanted for the artificial intelligence tool, and a trial was conducted on the sample model outside the scope of the research. Observations revealed that students had difficulty asking questions about artificial intelligence. At this point, it was shown that they could benefit from the sample keywords on the web page developed, and they were informed that they should ask short, simple questions (prompts) to the artificial intelligence and that they could get better answers in this way. A user manual and instructional videos were also distributed to ensure students' proficiency with the platform.

The instructional intervention lasted 2 weeks, with each of the three groups completing 4 h of theoretical and practical activities. To adhere to the planned ID, several steps were taken to reduce the likelihood of external resource use. The researchers provided consistent support across all groups and ensured that all C, SML, and SML + AI groups did not consult additional digital or print resources other than those provided to them. This was reiterated at the start of each session and included in the task guidelines. Participants in SML + AI were provided with clear, written instructions emphasizing that they should rely solely on ChatGPT's responses to complete their tasks. Throughout this process, the instructor continued to monitor the process in both SML and C with great care, and did not allow them to conduct quick knowledge checks through the internet or related documents. Additionally, the instructor ensured that all groups adhered to the timing of the tasks and directions. Interaction with the participants was kept to a minimum to avoid influencing their participation or reactions during the activities. Also, the feedback provided during the interventions on the questions was consistent, and no additional support was required. Following the experimental process, AAT and CLS were administered as posttests in all groups, and semistructured interviews were conducted with thtoe SML + AI group to gather qualitative feedback. Additionally, the AAT was readministered 5 months later to evaluate long‐term knowledge retention across all groups.

### 5.5 Data Analysis

Data analysis followed a mixed‐methods approach, encompassing quantitative and qualitative phases. First, the quantitative analysis techniques were performed to examine academic achievement, cognitive load, and learning retention related to anatomy instruction among the three groups. A two‐way ANOVA was used to analyze the pre‐ and posttest results of the AAT and CLS, while a one‐way ANOVA was used to assess learning retention. Descriptive statistics (mean and standard deviation) were calculated, with significance set at the *p* = 0.05 level. The LCD post hoc test identified mean differences, and all analyses were conducted using SPSS 27.0.

Second, the qualitative analysis focused on the SML + AI group's perspectives on anatomy education, employing content analysis to organize written responses into concepts and themes. During the initial coding phase, main categories and subcodes were developed to support the analysis, allowing previously unrecognized themes and codes to emerge. Rather than seeking “correct” answers, the analysis emphasized capturing participants' perspectives on their learning experiences with the SML + AI ID. All researchers independently reviewed the data to create open codes, which were collaboratively refined into categories and grouped into primary and secondary themes. Discrepancies were resolved through discussion to ensure reliability. Findings are reported as descriptive themes, with pseudonyms (e.g., S1, S2) used to maintain student anonymity.

## 6 Results

The findings obtained from the data collection tools applied to C, SML, and SML + AI groups within the scope of the research questions are presented below.

### 6.1 Research Question 1

The first research question was posed to evaluate if there was a significant difference between the C, SML, and SML + AI groups in terms of anatomy academic achievement. For this purpose, the AAT was administered to students in the groups before and after the implementation. Accordingly, it is observed that the anatomy achievement scores of students in all groups were almost similar before the anatomy course (${\bar{x}}_{c}$= 17.73, ${\bar{x}}_{\mathrm{SML}}$= 18.68, ${\bar{x}}_{\mathrm{SML}+\mathrm{AI}}$= 19.48). Following the anatomy instruction sessions conducted in groups, it is evident that the SML + AI group exhibited a noticeable increase in academic achievement compared to the other groups (${\bar{x}}_{c}$= 28.96, ${\bar{x}}_{\mathrm{SML}}$= 28.64, ${\bar{x}}_{\mathrm{SML}+\mathrm{AI}}$= 38.19).

A two‐way ANOVA was conducted to explore the mean differences in anatomy academic achievement across groups (C, SML, and SML + AI) or experiments (pre‐ and posttest), and the results are given in Table 1.

**TABLE 1:** Differences in achievement mean scores regarding group, experiment, and their interaction.

Source of interaction	Sum of squares	df	Mean squares	*F*	*p*	Partial eta squared (*η* ^2^)
Group	989.22	2	494.61	3.53	0.03	0.047
Experiment	6577.48	1	6577.48	47	0.000	0.246
Group × Experiment	570.69	2	285.34	2.04	0.13	0.028

It was found that there was a statistically significant difference between the anatomy achievement scores obtained by the C, SML, and SML + AI groups (*F* = 3.53; *p* < 0.05; *η* ^2^ = 0.047). It was also observed that the experimental design resulted in a significant difference in academic achievement scores (*F* = 47; *p* < 0.05; *η* ^2^ = 0.246). This finding shows that the C, SML, and SML + AI students' anatomy achievement scores differ regardless of the experiment. However, upon examining the effect size values contributing to the differentiation in achievement scores, it was revealed that the largest effect size originated from the experimental design (*η* ^2^ = 0.246). In order to see the specific source of the academic achievement differences among the groups (C, SML, and SML + AI) and between the experimental process (pre‐ and posttest), a post hoc test was conducted, and the results are shown in Table 2.

**TABLE 2:** Post hoc test results on academic achievement between group differences.

Group (*I*)	Group (*J*)	Mean difference (*I* − *J*)	Std. error	*p*
C	SML	−0.31	2.42	0.897
	SML + AI	−5.49*	2.3	0.018
SML	C	0.31	2.42	0.897
	SML + AI	−5.17*	2.4	0.033
SML + AI	C	5.49*	2.3	0.018
	SML	5.17*	2.4	0.033

* *p* < 0.05.

**FIGURE 3:**
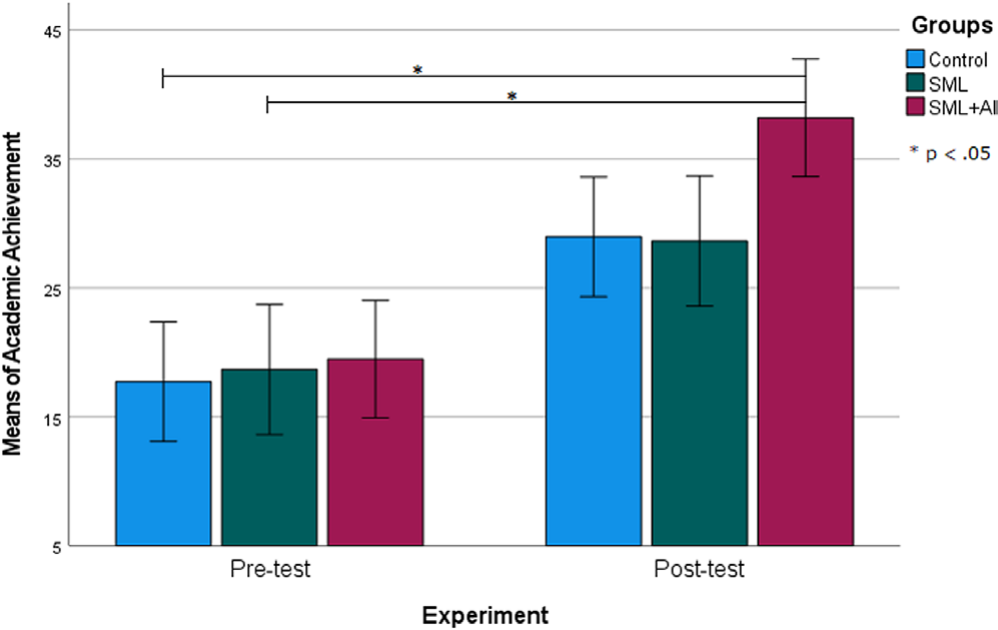
Academic achievement means differences between the groups over the experiment. Whiskers indicate ±1 standard deviation.

As shown in Table 2, a post hoc LCD test for multiple comparisons showed that the mean values of academic achievement scores differed significantly at the *p* < 0.05 level, confirming whether the SML + AI group exhibits superior posttest performance compared to both C and SML. In this context, it can be said that the participation of students in instructional activities through an SML + AI‐based experimental design had a greater impact on the differentiation of anatomy success (Figure 3).

### 6.2 Research Question 2

The second research question investigated if there was a difference between the C, SML, and SML + AI groups in terms of cognitive load. The CLS was systematically administered to students in groups before and after they completed the anatomy instructions. The cognitive load scores of students across all groups showed comparability before the experimental process (${\bar{x}}_{c}$= 5.85, ${\bar{x}}_{\mathrm{SML}}$ = 5.77, ${\bar{x}}_{\mathrm{SML}+\mathrm{AI}}$= 5.85). Following the anatomy course sessions, it is evident that the SML + AI displayed a notable decrease in cognitive load compared to the other groups (${\bar{x}}_{c}$= 5.38, ${\bar{x}}_{\mathrm{SML}}$ = 5.05, ${\bar{x}}_{\mathrm{SML}+\mathrm{AI}}$ = 4.56). A two‐way ANOVA test was conducted to determine whether the observed change in cognitive load scores was due to a particular source, and the analysis results are given in Table 3.

**TABLE 3:** Differences in the cognitive load scores regarding group, experiment, and their interaction.

Source of interaction	Sum of squares	df	Mean squares	*F*	*p*	Partial eta squared (*η* ^2^)
Group	4.49	2	2.24	1.24	0.29	0.017
Experiment	25.53	1	25.53	14.12	0.001	0.089
Group × Experiment	4.81	2	2.4	1.33	0.26	0.018

Although there was no statistically significant difference in cognitive load scores for the three groups (*F* = 2.24; *p* > 0.05; *η* ^2^ = 0.017), it was determined that the experimental design caused a significant difference in students' cognitive load structures (*F* = 14.12; *p* < 0.05; *η* ^2^ = 0.089). Also, the effect size value of the experimental process has the highest level, indicating that cognitive load differences between groups originat from the experimental design (*η* ^2^ = 0.089). In order to see the specific source of the cognitive load differences among the groups (C, SML, and SML + AI) and between the experimental phases (pre‐ and posttest), post hoc test was conducted, and the results are shown in Table 4.

**TABLE 4:** Post hoc test results on cognitive load between group differences.

Group (*I*)	Group (*J*)	Mean difference (*I* − *J*)	Std. error	*p*
C	SML	0.21	0.275	0.455
	SML + AI	0.41	0.261	0.117
SML	C	−0.21	0.275	0.455
	SML + AI	0.21	0.273	0.453
SML + AI	C	−0.41	0.261	0.117
	SML	−0.21	0.273	0.453

**FIGURE 4:**
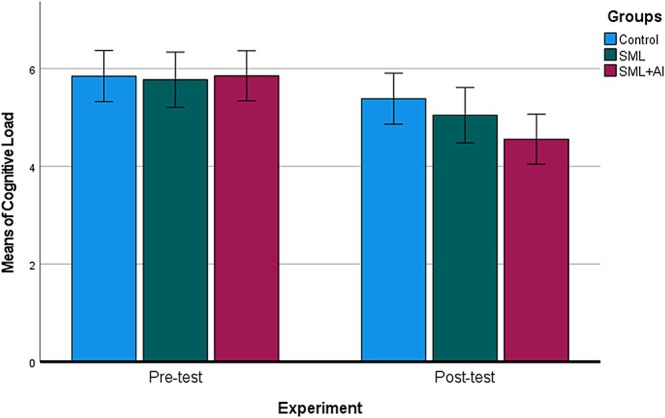
Cognitive load mean differences between the groups over the experiment. Whiskers indicate ±1 standard deviation.

As shown in Table 4, a post hoc LCD test for multiple comparisons showed that the mean values of cognitive load scores of all groups are not significant (*p* > 0.05). In consequence of the instructions provided throughout the experimental procedure, the cognitive load score of the SML + AI group was observed to decrease in comparison to the other groups; however, this reduction can be interpreted as indicating that no significant difference existed between the other groups themselves (Figure 4).

### 6.3 Research Question 3

The third research question was addressed to evaluate the anatomical knowledge retention among students following anatomy training. For this purpose, AAT has been administered again to students in the C, SML, and SML + AI. Accordingly, students in SML + AI have a higher retention level (${\bar{x}}_{\mathrm{SML}+\mathrm{AI}}$ = 49.37) than other groups (${\bar{x}}_{C}$ = 32.00; ${\bar{x}}_{\mathrm{SML}}$ = 33.91). Regarding the scores of the groups, a one‐way ANOVA was performed to compare the effect of the instructional process on retention between groups, and the analysis results are given in Table 5.

**TABLE 5:** One‐way ANOVA results on retention level.

	Source	Sum of squares	df	Mean square	*F*	*p*
Retention level	Between groups	4745.27	2	2372.63	12.02	0.000
	Within groups	14,208.11	72	197.33		
	Total	18,953.38	74			

As a result of the ANOVA test scores, there is a significant difference in retention between at least two groups *(F*_(2, 72)_ = 12.02; *p* < 0.05). To see the specific source of the retention differences among the groups (C, SML, and SML + AI) and between the experimental process (pre‐ and posttest), a post hoc test was conducted, and the results are shown in Table 6.

**TABLE 6:** Post hoc test results on retention between group differences.

Group (*I*)	Group (*J*)	Mean difference (*I* − *J*)	Std. error	*p*
C	SML	−0.74	4.96	0.988
	SML + AI	−20.25*	4.71	0.000
SML	C	0.74	4.96	0.988
	SML + AI	−19.51*	4.92	0.001
SML + AI	C	20.25*	4.71	0.000
	SML	19.51*	4.92	0.001

* *p* < 0.05.

**FIGURE 5:**
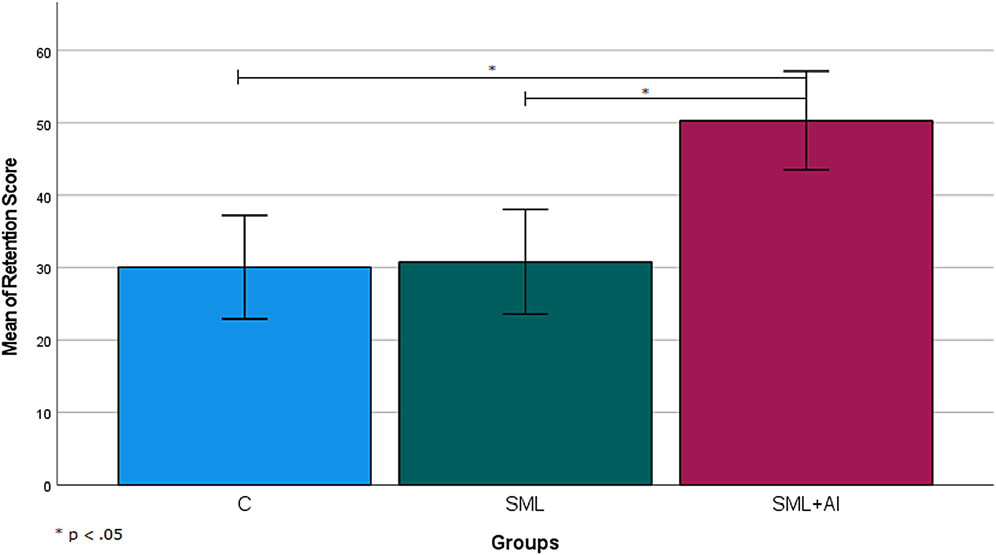
Retention mean differences between the groups over the experiment. Whiskers indicate ±1 standard deviation.

Based on the LCD post hoc test results, the source of this significance lay between SML + AI and the other groups (*p* < 0.05). In this context, the involvement of students in anatomical activities through SML + AI‐based experimental design led to higher retention of anatomical knowledge, as illustrated in Figure 5.

### 6.4 Research Question 4

**FIGURE 6:**
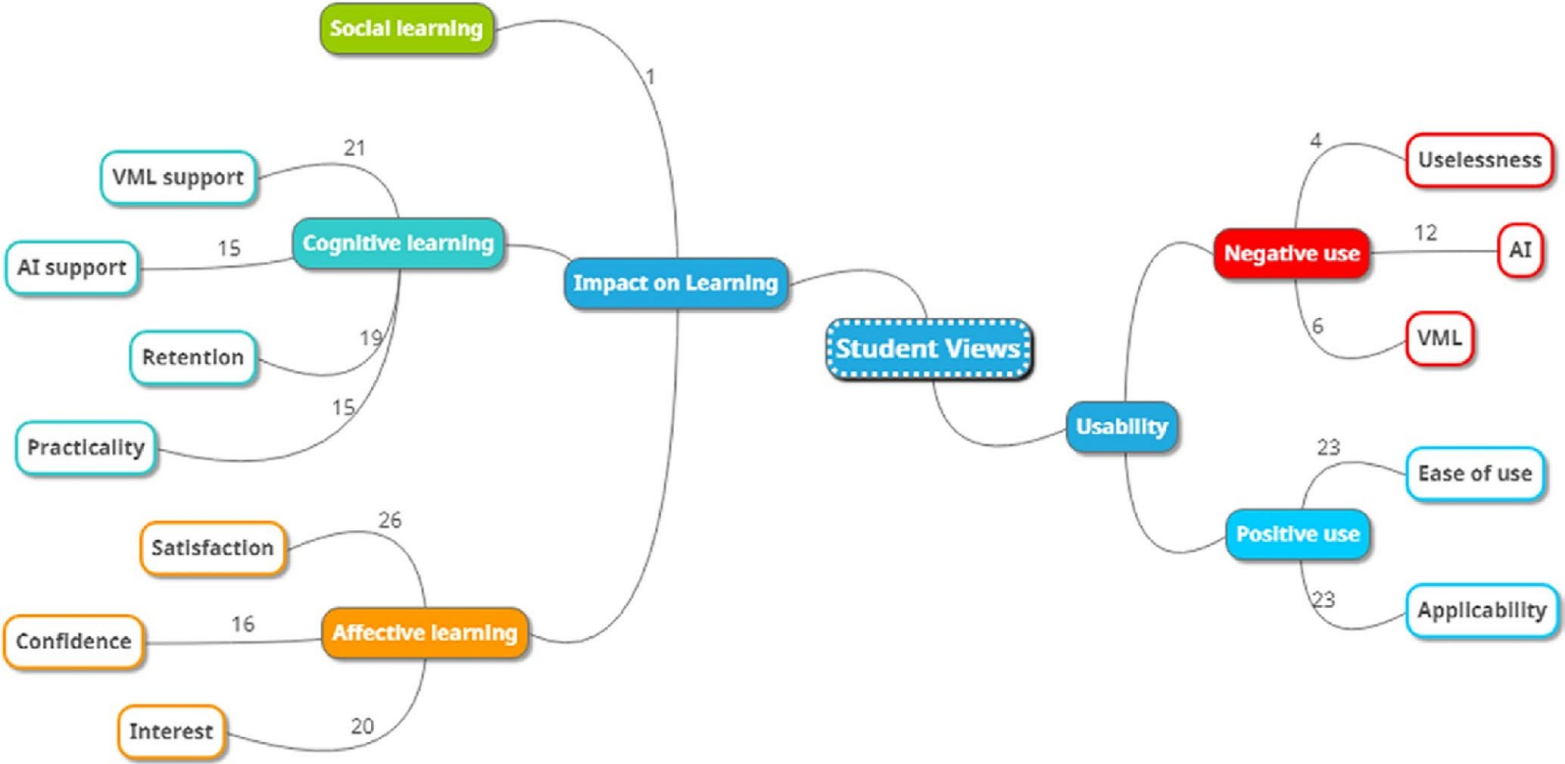
The themes and subthemes are determined based on student views.

The fourth research question was posed to identify the opinions of SML + AI students about the anatomy instructions in terms of the learning experiences through the experimental process. The views of the SML + AI students about ID were categorized into two themes: “usability” and “impact on learning” (Figure 6).

We categorized the opinions under the main theme of *usability*, considering both the positive and negative experiences reported during the lessons. Notably, a large number of students expressed positive views, which were grouped under the subthemes of *applicability* and *convenience*. Regarding the applicability of the instructional technologies used in the SML + AI group, student S12 highlighted the practicality of the simulation and ChatGPT tools by stating, “I think it is an applicable method for students because it is easy to use and free of charge.” Similarly, S7 commented, “I had no difficulty using the program; I think it is a very understandable and applicable system.” Students emphasized the usefulness of the instructional methods used in the lessons, particularly their ease of use. This is reflected in student S18's statement: “I do not think anything is challenging about it, anyone with simple computer usage knowledge can easily use this technique.”

It is noteworthy that a minority of student respondents provided negative evaluations of the combined use of simulations and artificial intelligence in practical activities. It is understood that these responses about the teaching methodology as unnecessary, concerns about AI, and issues with virtual manipulations. The majority of negative opinions regarding AI were technical, particularly, focusing on the feedback generated by ChatGPT. S21 stated that “In responding to our prompts, the AI frequently utilized terminology not typically associated with anatomical. The responses were comprehensive, they were phrased in a vernacular rather than a formal medical style.” Similarly, S19 noted “When we ask the same questions in different ways, it can give different answers. He answers the information more straightforwardly, not according to medical books. This was the most significant shortcoming.” Although it is understood that other negative opinions emerged due to the virtual manipulative designs, S23 described this situation as “Although the virtual manipulative part is useful, it can be made more comprehensive in terms of content.” In contrast, S10 believes that the teaching method is not necessary, cannot internalize the applied method with his opinion as “I think it is not a very necessary application. It is not like the methods we are used to.”

The responses under *i mpact on learning* were categorized into the subthemes of cognitive, affective, and social learning. Nearly all students emphasized that the SML applications played a crucial role in facilitating cognitive learning. S16 expressed this: “I had difficulty thinking about the subject in three dimensions because we could not always use the anatomy laboratory. In this respect, it supported my learning. Anatomical subject explanations became more concrete.” Similarly, S25 highlighted the importance of visual and spatial elements in learning supported by SML, as “It makes it easier to understand and memorize because we have the opportunity to examine 3D formations all the time and it appeals to visual memory.”

It is noteworthy that students evaluated the effectiveness of AI support on cognitive learning in both positive and negative ways. Many students who emphasized the facilitative role of AI support in learning stated that they no longer needed books and lecture notes. S22, who expressed a negative opinion, found the AI‐generated responses excessively long and sometimes irrelevant, although generally accurate, by remarking, “The answers it gives differ according to how the question is asked. It answers what normal people should know instead of medical ones, which confuses me. For example, it could not answer turuncus cerebri.” Beyond cognitive learning, students' opinions on the effectiveness of the ID on long‐term memory. S24 emphasized retention in their learning with the statement, “Since there is a different education method and the possibility of continuous access to visuals, it helped me keep the information in my memory longer.”

Learning outcomes such as satisfaction, self‐confidence, willingness, and interest can be part of affective learning. Compared to conventional instructional techniques, it was observed that anatomy subjects through instructional technologies within the scope of SML + AI aroused interest and enthusiasm in many students. For instance, S5, who experienced increased motivation towards the course, expressed his satisfaction by stating, “Since anatomy is a difficult course, the methods applied in this way increase my motivation and satisfaction.” Additionally, S7 emphasized the cognitive contribution of the instant feedback mechanism provided by AI support. It is possible to understand the positive effect of this situation on his self‐confidence towards the course from his statements: “Getting instant answers to my questions made me understand the subject quickly and therefore made me feel more confident.”

S19, who believed that social interaction contributes to social development, stated: “The laboratory setting and discussion of physical models facilitates a more socially interactive learning environment. Conversely, the virtual learning environment may diminish social interaction, potentially leading to technological dependence.” This reflects a negative evaluation of the teaching methodology implemented through SML + AI.

## 7 Discussion

ChatGPT, which has recently been used in various fields, including education (Sarker 2022), has been claimed to cause paradigm shifts in health sciences education (Sallam 2023). In the study in which we investigated the effect of ChatGPT‐supported virtual manipulative education environment on anatomy education, we found that this technique had positive effects on academic achievement, cognitive load, and retention of the course, and also, based on the opinions of the students, it may have positive effects on usability and retention, and adverse effects on the answers received, although few.

In the 20th century, with the advent of technological revolutions, educators, including those in medical education, have been experimenting with new teaching methods using computers, the internet, and other tools (Karim Qayumi and Qayumi 1999). In anatomy education, 3D interactive anatomy teaching platforms were the first steps in using computer‐based technology (Abdellatif et al. 2022). Although these new techniques have been tried, the debate on how to teach anatomy most effectively continues to be ongoing. Although cadaveric education has formed the basis of anatomy education for hundreds of years, this technique is considered to be outdated, costly, time‐consuming, and hazardous to health (Estai and Bunt 2016). In addition, students report that the curriculum is heavy and the lessons are boring and uninteresting (Potu et al. 2013; Estai and Bunt 2016). These negativities decrease their academic success and create problems that may affect their clinical years (Abdellatif et al. 2022). This literature result shows us that there is a need for methods that will facilitate anatomy education and enhance academic success. Our study results clearly show that the ChatGPT‐supported virtual manipulative training environment increases academic success in response to this need.

Health professionals should possess detailed knowledge of anatomy (Estai and Bunt 2016). Despite this necessity, students stated that anatomy is a challenging and complex course that relies heavily on rote memorization and requires a greater desire to learn. These compelling reasons lead students to require motivation while learning the course (Fitzgerald et al. 2008; Gaur et al. 2020; Johnson 2019). In line with the feedback we received from the students, we have seen that the applied technique aroused interest and desire for the lesson, thanks to its ability to provide instant feedback. At the same time, students also stated that their motivation to study increased.

The lack of motivation experienced by students in anatomy education increases cognitive load (Johnson 2019). In addition, the theoretical lecture given by the instructor in classical anatomy education is perceived as cognitively challenging by the student (Fitzgerald et al. 2008). It is essential to consider the new techniques integrated into anatomy education regarding cognitive load (Gaur et al. 2020). In studies evaluating the techniques applied in anatomy in terms of cognitive load, we can see that cognitive load decreases (Demir et al. 2023; Michalski et al. 2023), as well as studies that do not affect or increase cognitive load (Jamniczky et al. 2017; Birbara and Pather 2021; Vandenbossche et al. 2024). One of the reasons for the increase in cognitive load may be that more than one different information source is given, and the student has to divide his/her attention between various information sources. The new method should be well‐designed regarding cognitive load, and a pilot application should be carried out before the students (Sweller 2010; Birbara and Pather 2021). It is also essential to present the methods to the students as a single primary source (Mousavi et al. 1995). The study's design enabled the student to use virtual manipulatives and the artificial intelligence tools simultaneously. The methods used should be understandable and straightforward, especially for basic and complex courses such as anatomy. In the groups we evaluated in terms of cognitive load, we saw that the cognitive load of the SML + AI group decreased. Considering that students are not used to this new technique, this result may be significant for the applicability of the new technique.

It is known that various technological teaching methods have been incorporated into anatomy education to enhance future surgical competencies, preserve anatomical knowledge, and promote academic success (Estai and Bunt 2016; Patra et al. 2022). In the current century, students' learning habits have significantly improved; they utilize technological materials in addition to textbooks to obtain information (Owolabi and Bekele 2021). Since anatomy is a course based on visualization, it adapts well to technology (Sharma and Kumar 2021). For this reason, it has the potential to benefit from artificial intelligence (Chan and Zary 2019; Topol 2019). ChatGPT, as a virtual teaching assistant, is claimed to provide quick responses to information on medical topics and is an essential educational resource for students (Kung et al. 2023). It has been reported that it can answer questions about courses such as anatomy and histology quickly and understandably, and provide explanations (Chan and Zary 2019; Whalley et al. 2021; Denny et al. 2015). It also supports individual learning by providing access to information at any time. In support of this information, the students who participated in the study stated that using this educational technique was practical and helped to increase their retention of the course material by supporting their learning. In addition, we obtained results that statistically supported this finding in the retention test conducted 5 months after the first application. It would be useful to discuss the reasons for the differentiation between the groups as a result of cognitive load, in relation to the cognitive load theory. In line with the Cognitive Load Theory (Sweller 2010), cognitive load is formed as a result of various components, including intrinsic, extraneous, and germane load. In anatomy and health education, intrinsic cognitive load is considered naturally high due to the content's spatial complexity and abstract nature (Khalil et al. 2005). Accordingly, unless the designs of the instructional conditions differ significantly, perceived differences in total load are unlikely to be evident. From this point of view, it would not be wrong to say that the reason for the differentiation between the groups is due to the instructional structure directed at the groups. Although the mode of support differed (e.g., AI‐based versus traditional training), all participants were exposed to the same anatomical content and learning objectives. Accordingly, the reason for the decrease in the total load in the SML + AI group is likely due to the germane load delivered with GenAI support. As suggested by studies in anatomy education, the presence of guidance (e.g., through multimedia tools or intelligent tutoring systems) can reduce extraneous load without changing overall cognitive effort (Yammine and Violato 2015), thus leading to better outcomes without measurable changes in perceived load.

By combining pedagogical resources, effective learning is achieved when multimodal and system‐based approaches are integrated into the course, and this is also true for anatomy courses (Estai and Bunt 2016). The use of many educational theories with proven effectiveness in anatomy education is relatively low (Yachou et al. 2024). Teaching of basic medical sciences can be improved with the use of technology and media (Le and Prober 2018). ChatGPT, one of these technologies widely used in every field, has started to arouse interest in medical education. Although this technique is used in some courses (Lindqwister et al. 2021), we have yet to see any experimental studies in the literature that we could access, especially in anatomy education, that measure student success and satisfaction with ChatGPT, and gather student opinions. Although there are no studies on ChatGPT, some preliminary evaluations have guided us (Lee 2023; Abdellatif et al. 2022; Totlis et al. 2023; Lazarus et al. 2024; Li et al. 2021). The studies mostly asked questions, created questions (Leng 2024; Totlis et al. 2023; Mogali 2024; Ilgaz and Celik 2023), or used artificial intelligence in techniques such as VR and AR (Lazarus et al. 2024). ChatGPT have been reported as a valuable educational resource due to its numerous features, including the ability to provide information at any time and its interactive simulation potential (Lee 2023). When we asked the students about the use and applicability of the system, most gave positive answers. This easily applicable method can bring innovation to traditional teaching. The fact that very few students said ChatGPT was not necessary may be evidence for this idea.

However, some studies have claimed that ChatGPT provides insufficient and incorrect answers, particularly in response to anatomy‐related questions (Leng 2024; Totlis et al. 2023; Mogali 2024). We saw this deficiency in some of the feedback we received from the students. We have observed similar shortcomings through feedback received from students. As universally acknowledged, health sciences education requires a high level of accuracy. Without this, the lack of precise and complete information can lead to problems in students' clinical practice and may result in consequences that directly affect human lives. While existing literature demonstrating the widespread use of ChatGPT among medical students (Abdelhafiz et al. 2025; Park 2023), the accuracy of its responses, especially within the context of medical education, remains questionable. This is one of the main reasons we chose to incorporate ChatGPT into our study. What kind of outcomes can a widely used AI tool yield when implemented holistically and under the guidance of an educator? For this reason, this language model needs to be developed in a way that adapts to information and innovations, especially in medicine. Otherwise, it may cause information confusion for students. In addition, ChatGPT may give different or incomplete answers to the questions asked based on human use. It has been suggested that user manuals be created to prevent this (Leng 2024). When we looked at the literature, we saw that one of the essential features of ChatGPT is that it is designed in a way that can remember previously asked questions and self‐correct according to feedback. It is also claimed to be trained with a large data set (books, articles, internet content, etc.) (Fitria 2023). We think that integrating medical books and clinical experiences into ChatGPT can revolutionize medical education in terms of accessibility to information.

It is well known that the quality and diversity of input data used to train or develop artificial intelligence systems play a significant role in the outputs produced. During the preprocessing phase, examining the data, as well as during algorithm development and post‐processing, evaluating the output is essential to reduce biases (Nelson 2019). These biases are among the ethical concerns related to the use of artificial intelligence in medical education (Franco D'Souza et al. 2024). Another important ethical concern is ensuring the accuracy and reliability of the information. To ensure the correctness of information, it is important to encourage continuous feedback from both students and educators (Boscardin et al. 2024). Ongoing feedback will also enhance the quality of education provided by AI systems.

ChatGPT is an AI‐based large language model trained on large text datasets that can produce human‐like responses in multiple languages, including Turkish. It is a chatbot that can ask follow‐up questions, test definitions, and question assumptions (Sallam 2023). Especially with the pandemic, new perspectives have emerged in anatomy education. Although the theoretical courses were not a big problem, laboratory courses were unsuccessful during the pandemic (Ozen et al. 2022; Chang et al. 2022). From this point of view, it is essential to investigate distance and individual education methods, especially. Even if education is not entirely remote, directing students to individual learning will improve the quality of education.

## 8 Limitations and Future Research

There are some limitations in the study as follows:

– The fact that the research was conducted exclusively with students enrolled in a nursing department at a university. This sample is suitable for the study's focus on anatomy and nervous system education, but its generalizability to other health‐ and nonhealth‐related student populations is limited. Future research should aim to replicate the findings across diverse disciplines to strengthen external validity and explore variations in AI‐supported learning outcomes.
– In the context of anatomy, the nervous system was identified as a key area of interest in this study. The designed model's effectiveness should be examined on different anatomical systems.
– The testing technique was preferred to determine the anatomy academic achievement levels of the students. This approach provides data on learning outcomes, but may not fully capture students' abilities to apply anatomical knowledge in clinical contexts. Future research should consider incorporating various assessment methods to better evaluate participants' anatomical competence and learning transfer.
– ChatGPT 3.5 free version has been used throughout the experimental process, rather than more advanced models such as ChatGPT‐4.0, Claude, or Gemini. A potential limitation of the present study is the use of ChatGPT‐3.5. While these models may offer superior reasoning and domain‐specific accuracy, our focus was on the pedagogical aspects of engagement with widely accessible AI tools. Future research may benefit from comparative analyses that examine how different LLMs influence instructional quality and user experience across diverse educational tasks.
– In this study, cognitive load was assessed via a self‐reported type of cognitive load scale. We acknowledge this should be recognized as a limitation, and it is recommended that future research should include multidimensional measures (e.g., intrinsic, extraneous, and germane components) (Kalyuga 2011; Kühl et al. 2011).
– Virtual manipulations were limited on the web‐based interface systems called AnatomyTOOL and Shetchfab systems. Future studies should consider integrating more advanced virtual dissection tools, augmented reality (AR) or haptic‐enabled platforms that offer greater interactivity with AI.
– One of the most significant limitations of the study is the short duration of the intervention. The literature indicates a need for longer‐term experimental studies.
– The study was conducted in December 2023 using ChatGPT‐3.5. However, it is important to note that this version of the model was trained on data only up to September 2021. Therefore, its responses may not reflect the most recent developments or updates in anatomical knowledge and medical education.

## 9 Conclusion

Although cadaveric training is indispensable in anatomy education, educators are searching for engaging teaching tools in line with current trends (McLachlan et al. 2004). An urgent need is to integrate effective technological and creative teaching methods to support anatomy education into classical education (Longhurst et al. 2020; Saverino 2021). Although this instructional technique, designed by experts in the field, reduces cognitive load and improves academic performance, the inadequacy of ChatGPT's responses to questions has raised doubts about its current usability in anatomy education. In addition, despite the inadequacy of its responses to questions, its positive impact on learning also provides insight into the learning habits of today's students. Considering the rapid development of artificial intelligence tools, we believe that their integration into medical education will also progress quickly. Therefore, studies on artificial intelligence and medical education will continue to attract significant attention.

## Disclosure

Assurance that the manuscript is an original work, has not been published previously either in whole or in part, except in abstract form, and is not under consideration for publication by any other journal.

## Ethics Statement

The study's compliance with ethical principles was evaluated by the Social and Human Sciences Ethics Committee of Bursa Uludag University, which granted the ethical approval (numbered: E‐90661511‐000‐14464, dated 13/12/2023).

## Data Availability

The data that support the findings of this study are available from the corresponding author upon reasonable request.
